# Early enteral nutrition with exclusive donor milk instead of formula milk affects the time of full enteral feeding for very low birth weight infants

**DOI:** 10.3389/fnut.2024.1345768

**Published:** 2024-04-24

**Authors:** Min Wang, Xiaohui Gong, Lianhu Yu, Feifei Song, Dan Li, Qiaoling Fan, Ting Zhang, Xueming Yan

**Affiliations:** ^1^Department of Gastroenterology, Hepatology and Nutrition, Shanghai Children’s Hospital, Shanghai Jiao Tong University, School of Medicine, Shanghai, China; ^2^Institute of Pediatric Infection, Immunity and Critical Care Medicine, Shanghai Children’s Hospital, Shanghai Jiao Tong University, School of Medicine, Shanghai, China; ^3^Shanghai Donor Human Milk Bank, Shanghai Children’s Hospital, Shanghai Jiao Tong University, School of Medicine, Shanghai, China; ^4^Department of Neonatology, Shanghai Children’s Hospital, Shanghai Jiao Tong University, School of Medicine, Shanghai, China; ^5^Department of Pediatrics, Shandong University, Qilu Hospital, Jinan, Shandong, China; ^6^Shanghai Children’s Hospital, Shanghai Jiao Tong University, School of Medicine, Shanghai, China

**Keywords:** full enteral feeding, donor milk, formula milk, premature infants, enteral nutrition

## Abstract

This study investigated the effects of exclusive donor milk or formula in the first 7 days after birth, on the time to full enteral feeding, growth, and morbidity of adverse events related to premature infants. This was a retrospective study carried out from July 2014 to December 2019 at the Department of Neonatology of Shanghai Children’s Hospital. All infants with a birth weight < 1,500 g and a gestational age ≤ 32 who received exclusive donor milk or formula in the first 7 days after birth were included in this study. The time to full enteral feeding (defined as 150 mL/kg) in the donor milk group was significantly shorter than in the formula group (18 vs. 22 days, *p* = 0.01). Donated breast milk was also associated with a lower incidence of NEC (4.4 vs. 7%, *p* < 0.01), ROP (3.8 vs. 13.2%, *p* < 0.01), and culture-confirmed sepsis (11 vs. 22.6%, *p* < 0.01). Using donated breast milk instead of current formula milk for early enteral nutrition can shorten the time to full enteral feeding and reduce the incidence of NEC, ROP, and sepsis.

## 1 Introduction

In recent years, the number of premature infants has been increasing year by year with the changes in the environment and people’s lifestyles (1). It has been reported that prematurity accounts for more than 10% of new births each year worldwide and has become one of the leading causes of neonatal death. The survival rate of infants born extremely preterm (gestational age less than 28 weeks) was 62.3% in China (2). Approximately 15% of these preterm babies weigh less than 1,500 g, belonging to very low birth weight (VLBW) (3). Compared to full-term infants, preterm infants have less well-developed organs, weaker immune function, and a significantly higher incidence of neonatal necrotizing enterocolitis (NEC), sepsis, and other related complications. The incidence rate of sepsis varies from 20 to 40%, while the incidence of NEC is approximately 7% (4, 5). Breast milk contains all the nutrients and important antibodies needed for early infant growth, and it is important for improving feeding tolerance, reducing infections, promoting neurological development, and improving long-term prognosis (6–9). It has been suggested that VLBW infants who receive higher amounts of breast milk may have reduced morbidity and mortality (10–14). VLBW infants fed with their own mother’s milk have a shorter duration of full enteral feeding and 6–10 times lower NEC incidence (15–18).

After birth, nutritional feeding should be started as soon as possible to enhance enteral feeding tolerance, preferably starting with colostrum, which has a high level of anti-infective properties. However, most preterm infants do not have access to parental breastfeeding during their hospital stay, especially during the first hours of life. Even if the mother starts pumping as soon as possible, she will not provide enough breast milk within 3 days of birth. In recent years, breast milk banks have emerged (19). Qualified donor breast milk has become an important source of enteral nutrition for preterm infants due to the similar nutritional composition and functions as mother’s milk, greatly satisfying the demand for early breastfeeding of preterm infants (20, 21). Breast milk banking has changed the feeding pattern of preterm babies to some extent (22).

Our department has established a breast milk bank since June 2016 to provide breastfeeding support to hospitalized premature infants in the neonatal department and advocates the early use of donated breast milk for enteral nutrition (mother’s milk is unavailable). In this article, we investigate the effect of using exclusive donor breast milk or formula in the first 7 days after birth on the time of full enteral feeding, growth, and morbidity of adverse events related to premature infants.

## 2 Materials and methods

### 2.1 Design and setting

This was a retrospective study on the effect of donor milk feeding within 7 days after birth on preterm infants. Preterm infants with gestational age ≤ 32 weeks or birth weight < 1,500 g admitted to the neonatal intensive care unit (NICU) of Shanghai Children’s Hospital from July 2014 to December 2019 were retrospectively included in the study. Inclusion criteria: admission within 24 h after birth, hospitalization for more than 2 weeks, and exclusive donor milk or formula feeding within 7 days after birth. Exclusion criteria included dysplasia of the digestive tract, severe congenital heart disease (excluding atrial septal/ventricular septal defect, patent ductus arteriosus, and patent foramen ovale), systemic metabolic diseases, mixed feeding or exclusive mother milk breastfeeding within 7 days after birth.

Infants received exclusive pasteurized donor milk or preterm formula (Nestle preNAN) during the first 7 days of life. After 7 days, infants received their mother’s breast milk if their own mother’s milk was available. After full enteral feeding, some infants in the donor milk group may be fed with preterm formula, as donated breast milk should be used preferentially for low gestational age infants. The numbers for infants with full enteral feeding in the donor milk group and formula group were 175 and 204, respectively. All parents were informed that their infant would receive donor milk and provided informed consent. This study has been approved by the Ethics Committee of Shanghai Children’s Hospital.

### 2.2 Standardized feeding regimen

The enteral feeding protocol for preterm infants weighing less than 1,500 g follows the guidelines published by the American Academy of Pediatrics and the European Society of Pediatric Gastroenterology, Hepatology, and Nutrition. All infants with very low birth weight received a gastric catheter during the first hour of life. At the same time, enteral nutrition (EN) was started within 24 h of birth in clinically stable preterm infants. Enteral feeding was advanced by approximately 20 mL/kg/day based on the infant’s feeding tolerance and total enteral feeding was defined as 150 mL/kg of enteral intake (23). If infants were breastfed, human milk fortifier (PreNAN, Nestle) was added at 100 mL/kg/day of EN, first at half-strength for 48 h and then at full strength. Once total enteral nutrition was achieved, parenteral nutrition (PN) was discontinued and central venous catheterization was removed if there was no need for continuous intravenous medication. During the transition phase, a “nutrition-based” transition program was implemented, aimed at maintaining target kcal and protein intakes. Parenteral glucose, amino acid, and lipid prescriptions were adjusted daily to maintain a goal energy intake of 100–120 kcal/kg/day (PN + EN) and protein intake of >3 g/kg/day, with parenteral lipids providing ≤50% of the parenteral energy. Serum glucose and triglycerides were maintained within acceptable limits.

### 2.3 Data collection and definitions

Basic information and clinical data were collected from the electronic medical record system. Basic information collected included gender, gestational age, birth weight, and mode of birth. Clinical data included time to start oral feeding, time to reach full enteral feeding, Apgar score, amount of enteral nutrition at 14 days, hospital stay, and weekly weight. The main complications of premature infants include NEC, intracranial hemorrhage (grade 3–4), bronchopulmonary dysplasia (BPD), late-onset sepsis (LOS), and the retinopathy of prematurity (ROP). The diagnostic and grading criteria for BPD were based on the definition of Jensen in 2019 (24). Respiratory support is still required at the corrected age of 36 weeks and the severity of respiratory support is graded based on the situation. NEC was defined using Bell staging criteria, stage 2 or greater (25). ROP is diagnosed based on fundus screening. Sepsis was defined as a positive blood culture and a C-reactive protein level>10 mg/L (4). C-reactive protein levels in patients’ serum were measured using the QuikRead go instrument with the QuikRead go hsCRP kit (Highly Sensitive Particle Enhanced Turbidimetric Method).

### 2.4 Statistical methods

The data were statistically analyzed using SPSS 25.0 statistical software. Normally distributed data were expressed as mean ± standard deviation (*x̄* ± s), and two-sample *t*-tests were used to compare the two groups. Non-normally distributed data were expressed as medians (interquartile spacing) [M (P25, P75)], and the Wilcoxon test was used for comparison between groups. The enumeration data were expressed as a percentage (%), and comparisons between groups were using the χ^2^ test or Fisher exact probability method. *p* < 0.05 indicated a statistically significant difference. Adjusted OR and its confidence intervals (95% CI) were determined using a logistic regression model and a value of *p* less than 0.05 was considered significant. Multivariate logistic analysis was adjusted with gestational age, sex, birth weight, mode of birth, antibiotics, antenatal steroids, and Apgar score at 5 min.

## 3 Results

### 3.1 Characteristics of the sample

A total of 394 preterm infants fulfilled the study criteria. Two hundred and twelve (53%) infants received exclusive formula and 182 (47%) were fed exclusive donor milk during the first 7 days of life. There were no statistically significant differences in birth weight, gestational age, sex, birth way, multiple birth rate, 5 min Apgar score, small for gestational age (SGA), maternal antibiotics use, and maternal steroid use between the two groups (*p* > 0.05). As shown in Table 1.

**Table 1:** Characteristics of study infants.

	Formula group	Donor milk group	*p*- value
*N*	212	182	
Birth weight (g)	1,214 ± 345	1,183 ± 296	0.53
Gestational age (w)	29.3 ± 2.0	29.1 ± 2.8	0.91
Male sex (%)	124 (58.5%)	93 (51.1%)	0.14
Cesarean section (%)	154 (72.6)	129 (70.9)	0.70
multiple births (%)	63 (29.7)	65 (35.7)	0.21
Apgar score at 5 min < 7 (%)	41 (19.3)	39 (21.4)	0.61
SGA (%)	38 (17.9)	23 (12.6)	0.15
Maternal antibiotics (%)	150 (70.7)	119 (65.4)	0.25
Maternal steroids (≥1 does) (%)	156 (73.6)	118 (64.8)	0.06

SGA, Small for gestational age.

### 3.2 Enteral feeding and growth

A total of 15 children in both groups died before reaching total enteral nutrition (eight in the formula group and seven in the donor milk group) and were therefore not included in the calculation of the time to reach full enteral feeding. The causes of death can be found in Supplementary Table S1. As expected, enteral feeds were initiated within 24 h. The amount of enteral nutrition on day 14 is 111.2 ± 13.4 in the formula group and 116.8 ± 17.2 in the donor milk group. Infants in the donor milk group achieved full enteral feeding 5 days earlier than those in the formula group, with a median of 13 days [interquartile range (IQR) 16–21], compared to a median of 18 days (IQR 22–28, *p* = 0.01) in the formula group. Infants in the donor milk group stayed 7 days shorter in the NICU than infants in the formula group, with a median of 50 days (IQR 47–53, *p* = 0.01) vs. a median of 43 days (IQR 40–47, *p* = 0.12).

There was no statistically significant difference in birth weight, 7-day weight, and 14-day weight between the two groups, but there was a difference in the discharge weight. Infants in the donor milk group weighed 2,532 ± 823 g and the weight in the formula group was 2,205 ± 754 g (*p* = 0.04). The absolute weight changes in individual birth weights were shown in Supplementary Figure S1. The weight of both groups of infants did not show a significant decrease in the first 7 days, but both showed a significant increase after 14 days. The weight gain at discharge was different between the two groups, with the formula group showing a greater increase. The difference in discharge weight may be related to the later discharge of the formula group (Table 2).

**Table 2:** Enteral feeding and body growth of the whole population.

	Formula group	Donor milk group	*p*-value
	*N* = 212	*N* = 182	
Onset of feeds (h)	22 (16–30)	18 (13–27)	0.45
Amount enteral feeding on day 14 (mL/kg)	111.2 ± 13.4	116.8 ± 17.2	0.33
Full enteral feeding (d)	18 (9–108)	13 (10–98)	0.01
Hospitalized days (d)	50 (9–145)	43 (1–142)	0.12
Birth weight (g)	1,214 ± 345	1,183 ± 296	0.53
Weight day 7 (g)	1,182 ± 355	1,217 ± 305	0.69
Weight day 14 (g)	1,473 ± 468	1,391 ± 414	0.28
Weight at discharge (g)	2,532 ± 823	2,205 ± 754	0.04
Died before reached full enteral feeding	8 (3.8%)	7 (3.8%)	0.84

### 3.3 Morbidities related to premature infants

In the whole population, 17 infants (8%) in the formula group developed NEC compared to six infants (3.3%) in the donor milk group (*p* = 0.04). Forty-eight infants (22.2%) in the formula group had culture-proven sepsis compared to 20 infants (11.5%) in the donor milk group (*p* < 0.01). Despite the differences in infectious outcomes, including NEC and culture-proven sepsis, we observed no differences in the rates of intubation, bronchopulmonary dysplasia, or intraventricular hemorrhage between the two groups (Table 3). In addition, the incidence of retinopathy of prematurity (ROP) significantly decreased in the donor milk group. After adjusting for the relevant confounders, exclusively formula feeding (OR 3.48, 95% CI 2.13–8.06, *p* = 0.04) in the first 7 weeks was significantly associated with a higher incidence of NEC, compared with the donor milk feeding. Similarly, exclusively formula feeding in the first 7 weeks was independently associated with an increased risk of ROP (OR 3.95, 95% CI 0.78, 9.05, *p* < 0.01) or culture-proven sepsis (OR 2.53, 95% CI 1.18–7.48, *p* < 0.01) compared with the donor milk feeding.

**Table 3:** Outcomes of different groups.

	Formula group	Donor milk group	OR^*^ (95% CI)	*p*-value
*N*	212	182		
Intubated	45 (21.2)	41 (22.5)	1.04 (0.31–3.03)	0.76
NEC	17 (8%)	6 (3.3%)	3.48 (2.13–8.06)	0.04
IVH III + IV	19 (9%)	11 (6%)	0.12 (0.02, 0.90)	0.62
BPD	33 (15.6%)	23 (12.6%)	0.16 (0.05–0.36)	0.41
ROP	28 (13.2%)	7 (3.8%)	3.95 (0.78, 9.05)	<0.01
Culture-proven sepsis	47 (22.2%)	21 (11.5%)	2.53 (1.18–7.48)	<0.01

Multivariate logistic analysis was adjusted with gestational age, sex, birth weight, mode of birth, antibiotics, antenatal steroids, and Apgar score at 5 min. The Donor milk group was the reference group. NEC, Necrotizing enterocolitis; IVH, Intraventricular hemorrhage; BPD, Bronchpulmonary dysplasia; ROP, Retinopathy of prematurity; OR, Odds ratio; and CI, Confidence interval. ^*^Multivariate analysis with adjusted OR using logistic regression.

Further, we grouped preterm infants according to gestational age (GA) <28 weeks and >28 weeks to observe whether early breast milk feeding would be beneficial for extremely premature infants, as shown in Table 4. We found 241 premature infants (61.2%) with GA >28 weeks and 153 premature infants (38.8%) with GA<28 weeks. The mobility of almost all adverse events was increased in preterm infants with GA<28 weeks compared to GA> 28 weeks, except for the donor milk group where there was a decrease in the incidence of NEC and sepsis. It can be found that early feed with donor milk has a more pronounced protective effect on preterm infants younger than 28 weeks in terms of the occurrence of NEC, ROP, and sepsis.

**Table 4:** Prevalence of preterm infant comorbidities in different gestational weeks.

	GA > 28 weeks (*N* = 241)			GA < 28 weeks (*N* = 153)		
	Formula group	Donor milk group	*p*-value	Formula group	Donor milk group	*p*-value
*N*	140	101		72	81	
Intubated	25 (17.9%)	19 (18.8%)	0.85	20 (27.8%)	22 (27.2%)	0.93
NEC	9 (6.4%)	4 (4%)	0.40	8 (11.1%)	2 (2.5%)	0.03
IVH III + IV	8 (5.7)	2 (2)	0.15	11 (15.3)	9 (11.1)	0.45
BPD	21 (15)	12 (11.9)	0.49	12 (16.7)	11 (13.6)	0.59
ROP	17 (12.1)	3 (3)	0.11	11 (15.3)	4 (4.9)	0.03
Culture-proven sepsis	29 (21)	12 (11.9)	0.07	18 (25)	9 (11.1)	0.02

NEC, Necrotizing enterocolitis; IVH, Intraventricular hemorrhage; BPD, Bronchpulmonary dysplasia; ROP, Retinopathy of prematurity.

### 3.4 Feeding at discharge

Preterm infants received exclusive formula or donor milk feeding within 7 days after birth. After 7 days, infants received donor milk or formula until their mother’s breast milk was available. If their mother’s breast milk was unavailable, they received donor milk or formula until they were discharged from the hospital. 26.4% of infants in the donor milk group were still fed—donor milk at discharge. 47.7% of infants in the formula group and 62.1% in the donor milk group received their mother’s own milk at discharge. The type of milk at the start of enteral nutrition did influence the type of feeding at discharge (Table 5).

**Table 5:** Type of feeding at discharge.

	Formula group	Donor milk group	*p*-value
	*N* = 212	*N* = 182	
Mother’s own milk	58 (27.4%)	63 (34.6%)	0.12
Donor milk	0	16 (8.8%)	<0.01
Formula	97 (45.8%)	34 (18.7%)	<0.01
Mother’s own milk + Donor milk	0	22 (12.1%)	<0.01
Mother’s own milk + Formula	43 (20.3%)	28 (15.4%)	0.21
Donor milk + Formula	0	10 (5.5%)	<0.01
Died before discharge	14 (6.6%)	9 (4.9%)	0.48

## 4 Discussion

In this retrospective study, we show that the intake of donor human milk during the first 7 days of life is associated with shorter full enteral feeding time and decreased morbidity of adverse events related to premature infants. The results of this study are consistent with the findings of previous studies, which showed that compared with formula, donated breast milk could significantly reduce the incidence of NEC and the time of full enteral feeding (26–29). We further substantiate and expand the pool of evidence suggesting a relationship between the donated breast milk the premature infants received and the lower risk of ROP or sepsis. In addition, we use donated breast milk exclusively within 7 days after birth to better illustrate the role of donated breast milk in early enteral nutrition for premature infants. These studies demonstrated that human milk intake is particularly important in the first few days of life. Feeding preterm infants with donor breast milk (rather than formula when the mother’s breast milk is not available) is associated with shortened time to full enteral feeding and reduced incidence of NEC, ROP, and sepsis. And it has a more pronounced protective effect on preterm infants with GA<28 weeks.

Breast milk is rich in immune active factors and low osmotic pressure has a good protective effect on the fragile intestinal barrier of very/ultra preterm infants. Donor milk has also been shown to promote feeding tolerance. Meta-analyses have shown that donor milk reduces the incidence of feeding intolerance compared to formula for preterm infants (30). An international survey of enteral feeding practices for very low birth weight infants also found that wards with donated breast milk sources can better establish total enteral feeding (31). As a result, the American Academy of Pediatrics recommends donor milk as a supplement or replacement for very low birth weight infants when parental breast milk is insufficient or contraindicated (32). The emphasis on donor milk feeding in the standardized enteral feeding protocol developed in conjunction with the availability of a breast milk bank in our hospital has contributed to a significantly higher rate of early donor milk use in very low birth weight infants, which may have been an important reason for the shortened time to achieve total enteral feeding.

In our study, we generally started enteral feeding within 24 h. Previous studies have found that the structural and functional integrity of the gastrointestinal tract in preterm infants is closely related to early enteral feeding. Postnatal fasting can cause thinning of the intestinal mucosa, flattening of the villi, and translocation of intestinal bacteria (33). Therefore, delayed enteral nutrition may be detrimental (34). Although early enteral feeding is a low-calorie, low-volume feeding, it facilitates the maintenance of higher gastrointestinal hormone levels and promotes continued maturation of intestinal motility, microbiome development and gastrointestinal function in preterm infants, thus improving tolerance of early enteral feeding (35–39). Therefore, the early use of donor breastfeeding advances the time of initiation of enteral feeding and facilitates the establishment of enteral nutrition.

This study found that early exclusive donor breastfeeding was associated with a reduced incidence of NEC, sepsis, and ROP in preterm infants. Many studies have also confirmed that donated breastfeeding can reduce the incidence of nosocomial infection, sepsis, and NEC (29, 40, 41). Quigley et al. (14) found that donor milk reduced NEC and helped improve neurodevelopment in a study of 1809 preterm infants fed with donor milk and formula. Villamor-Martínez et al. (42) also showed that donor milk feeding may prevent bronchopulmonary dysplasia. The neurodevelopmental benefits of donor breast milk for preterm infants are now quite clear, with meta-analyses showing that breastfed infants have higher levels of cognitive development compared to formula-fed infants and that the neurodevelopmental system of preterm infants is stimulated by certain nutrients in breast milk. In addition, the improved growth, health, and development can be attributed to lower inflammation among breast milk fed infants. Konnikova et al. found that the anti-inflammation and immunomodulating components of breast milk help to protect the preterm infant against infections, meningitis, and late-onset sepsis (43–45). Konnikova et al. found that breast milk can reduce the inflammatory condition of the intestine, thereby reducing NEC. They have shown that tryptophan and bifidobacterium in breast milk produce a metabolite response that acts as an anti-inflammatory by inhibiting aryl hydrocarbon receptor transcription factors that stimulate the IL-8 response (46). Breast milk contains many protective anti-inflammatory components that prevent excessive inflammation until the infant can develop its mature anti-inflammatory mechanisms.

In previous clinical work, we have not focused specifically on the relationship between donor breastfeeding and retinopathy of prematurity. In this study, we specifically observed that donated breast milk was more beneficial in reducing the incidence of retinopathy of prematurity in preterm infants. These results were consistent with existing research findings. Hylander et al. (47) showed that after excluding other confounding factors, breastfed very low birth weight infants had a lower incidence of neonatal retinopathy than formula-fed infants, suggesting that breastfeeding is also important for the neurological development of preterm infants. Another study found that any amount of human milk intake is strongly associated with protection from all ROP and severe ROP (48). They hypothesized that the antioxidant properties of breast milk and high levels of dodecahexaenoic acid are the reasons for the protective effect on ROP.

The occurrence of increased NEC, sepsis, or ROP with preterm formula may be related to the following reasons. Preterm formulas do not retain the immune components and active substances found in breast milk, such as lactoferrin, lysozyme, lipase, immunoglobulins, cytokines, and oligosaccharides, which promotes the development of bodies, helps with the maturation of the infant’s immune system (10, 43) and protects against infections. The composition of preterm formulas affects milk osmolality and the renal load, the latter may be a further factor in determining fluid intakes in preterm infants with limited renal concentration ability and excretory capacity (49, 50), thus affecting the water-electrolyte balance of the preterm infants. Furthermore, the osmotic pressure of milk affects intestinal permeability and inflammation, which can cause incomplete digestion of formula and increase the incidence of NEC (51, 52).

It is interesting to note that donating milk on admission increases the proportion of human milk feeding at discharge (62.1 vs. 47.7%), indicating that donating milk affects the feeding type at discharge. This is related to the promotion of breastfeeding through written, online, and social propaganda since the establishment of our breast milk bank, as well as the implementation of hospital breastfeeding guidance, emphasizing the importance of breast milk for the growth and development of premature infants and newborns.

There are limitations to this study. It is a single-center retrospective study, which has inherent limitations except for a small sample size. The data are influenced by the level of detail in medical records. We therefore selected indicators for analysis that are present in medical records.

## 5 Conclusion

In this study, we show that the intake of exclusive donor milk during the first 7 days of life is associated with shorter full enteral feeding time and decreased morbidity of adverse events related to premature infants, illustrating the importance of early donor milk feeding.

## Data availability statement

The raw data supporting the conclusions of this article will be made available by the authors, without undue reservation.

## Ethics statement

The studies involving humans were approved by Ethics Committee of Shanghai Children’s Hospital. The studies were conducted in accordance with the local legislation and institutional requirements. Written informed consent for participation in this study was provided by the participants’ legal guardians/next of kin.

## Author contributions

MW: Data curation, Methodology, Software, Writing – original draft. XG: Resources, Validation, Writing – review & editing. LY: Data curation, Methodology, Software, Writing – original draft. FS: Data curation, Software, Writing – review & editing. DL: Data curation, Formal analysis, Investigation, Writing – review & editing. QF: Resources, Validation, Writing – review & editing. TZ: Writing – review & editing. XY: Validation, Supervision, Visualization, Writing – review & editing.
