# Supplementary material for: Early enteral nutrition with exclusive donor milk instead of formula milk affects the time of full enteral feeding for very low birth weight infants
Source: Front Nutr. 2024 Apr 24;11:1345768. doi: 10.3389/fnut.2024.1345768 (PMC11076758; doi:10.3389/fnut.2024.1345768)
Supplement: Supplementary file 1 [file Table_1.DOCX]

Table S1. Causes of preterm infant mortality.

|  | Formula group | | | Donor milk group | | |
| --- | --- | --- | --- | --- | --- | --- |
|  | Total  N=14 | Died before full enteral feeding  N=8 | Died after full enteral feeding  N=6 | Total  N=9 | Died before full enteral feeding  N=7 | Died after full enteral feeding  N=2 |
| RDS | 1 | 1 | 0 | 0 | 0 | 0 |
| sepsis | 4 | 3 | 1 | 3 | 3 | 0 |
| NEC | 4 | 2 | 2 | 2 | 1 | 1 |
| IVH | 3 | 1 | 2 | 2 | 1 | 1 |
| BPD | 1 | 0 | 1 | 0 | 0 | 0 |
| Meningitis | 0 | 0 | 0 | 1 | 1 | 0 |
| Pneumorrhagia | 1 | 1 | 0 | 1 | 1 | 0 |

There were 14 deaths in the formula group, 8 died before reaching full enteral feeding and 6 after full enteral feeding. In the donor milk group, there were 9 deaths, 7 died before reaching full enteral feeding and 2 after full enteral feeding. The specific causes of death are shown in Supplementary Table 1 (Table S1).
