# Supplementary figures and images for: Early enteral nutrition with exclusive donor milk instead of formula milk affects the time of full enteral feeding for very low birth weight infants
Source: Front Nutr. 2024 Apr 24;11:1345768. doi: 10.3389/fnut.2024.1345768 (PMC11076758; doi:10.3389/fnut.2024.1345768)

Figure S1. Absolute weight differences from birth weights on day 7, day14 and discharge.

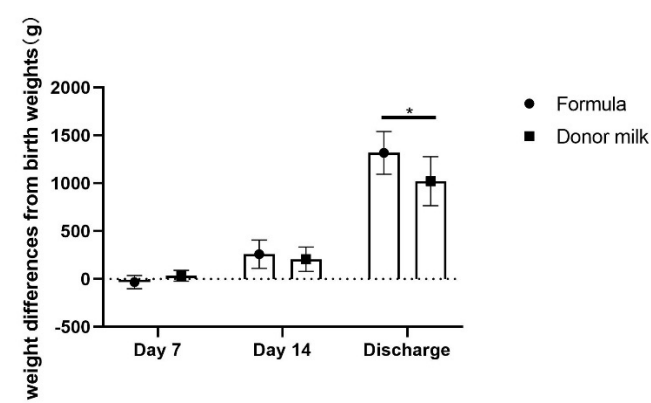

Supplement: Supplementary file 2 [file Image_1.pdf]
